# Comparative Toxicity and P450-Mediated Detoxification of Flonicamid in *Lygus lineolaris* and *Lygus hesperus*

**DOI:** 10.3390/insects16080757

**Published:** 2025-07-23

**Authors:** Yuzhe Du, Shane Scheibener, Yu-Cheng Zhu, Calvin Pierce, Omaththage P. Perera, Maribel Portilla

**Affiliations:** 1 Southern Insect Management Research Unit, Agriculture Research Service, United States Department of Agriculture, 141 Experiment Station Road, Stoneville, MS 38776, USA; shane.scheibener@usda.gov (S.S.); calvin.pierce@usda.gov (C.P.); op.perera@usda.gov (O.P.P.); maribel.portilla@usda.gov (M.P.); 2 Pollinator Health in Southern Crop Ecosystems Research Unit, Agriculture Research Service, United States Department of Agriculture, 141 Experiment Station Road, Stoneville, MS 38776, USA

**Keywords:** tarnished plant bug, western tarnished plant bug, flonicamid, toxicity, P450

## Abstract

The tarnished plant bug, *Lygus lineolaris* (TPB), (Palisot de Beauvois), and the western tarnished plant bug (WTPB), *Lygus hesperus*, Knight, are major agricultural pests that cause significant damage to a wide range of crops in the southeastern and southwestern United States. Flonicamid (commercial name: Carbine 50WG) is generally effective against various sap-feeding pests, including both *L. hesperus* and *L. lineolaris*. This study evaluated the toxicity of flonicamid on third-instar nymphs and adults of both *Lygus* species under laboratory conditions. Two bioassay methods were used: spray application to assess both contact and oral toxicity, and dipping to evaluate oral toxicity. Results showed that *L. hesperus* was significantly more susceptible to flonicamid than *L. lineolaris* across both bioassay methods. While no significant differences in toxicity were observed between spray and dipping assays, third-instar nymphs exhibited significantly higher sensitivity than adults in both species. The addition of piperonyl butoxide (PBO), a known inhibitor of cytochrome P450-monooxygenases (P450s), significantly enhanced the toxicity of flonicamid, suggesting that P450 enzyme plays a critical role in its detoxification. Sublethal exposure to flonicamid also induced increased P450 activity in both species. These findings provide valuable insights into the differences in susceptibility between *L. lineolaris* and *L. hesperus* to flonicamid and indicate that P450-mediated detoxification is critical for flonicamid metabolism. Such insights are valuable for early resistance monitoring and optimizing flonicamid application in integrated pest management programs.

## Simple Summary

This study investigated the toxicity of flonicamid against two major crop pests: the tarnished plant bug (*Lygus lineolaris*) and the western tarnished plant bug (*Lygus hesperus*). Laboratory bioassays revealed that *L. hesperus* was more susceptible to flonicamid than *L. lineolaris*. In both species, third-instar nymphs exhibited higher sensitivity than adults, regardless of whether a spray and dipping bioassay method was used. Furthermore, the presence of piperonyl butoxide (PBO), a cytochrome P450 inhibitor, significantly enhanced flonicamid toxicity, suggesting the involvement of P450 enzymes in its detoxification. The findings underscore the need to consider *Lygus* species- and stage-specific differences when incorporating flonicamid into integrated pest management strategies.

## 1. Introduction

The tarnished plant bug (*Lygus lineolaris*, TPB) (Palisot de Beauvois), (Hemiptera: Miridae) and the western tarnished plant bug (*Lygus hesperus*, WTPB) Knight, (Hemiptera: Miridae) are among the most economically important mirid pests in the United States. These pests cause severe damage to a wide variety of crops, including cotton, alfalfa, strawberry, lentil, safflower, and various fruit, vegetable and fiber crops. *L. hesperus* is particularly problematic in western regions such as California [1], Arizona [2] and the Texas High Plains [3], while *L. lineolaris* is the most destructive pest of cotton (*Gossypium hirsutum* L.) in the Mid-south United States [4]. Both *Lygus* species cause substantial economic loss in cotton by feeding on developing flower buds, resulting in square loss and the destruction of potential fruiting structures [4,5]. Their mobile and polyphagous feeding habits enable them to exploit various crop and non-crop habitats, further exacerbating their impacts on agricultural systems [6]. Despite decades of implementing chemical, biological and cultural crop protection, insecticide resistance has emerged in both *L. lineolaris* and *L. hesperus* populations. In particular, *L. lineolaris* control has shown increasing resistance to pyrethroids, organophosphates (OPs), carbamates and neonicotinoids over the past four decades [7]. The overuse of these insecticides has reduced their efficacy and necessitated the development of new chemistries to manage resistant populations and mitigate resistance development [8].

Flonicamid, a systemic insecticide belonging to the pyridinecarboxamide group, was discovered in 1992, commercially launched in 2005, and is currently registered for pest management in over 40 countries [9,10]. By 2018, its market value reached $55 million, representing approximately 0.28% of the global insecticide market [10,11]. Flonicamid is highly selective and shows excellent efficacy against Hemipteran pests including aphids, whiteflies, thrips, leafhopper and planthoppers, while exhibiting low toxicity to beneficial insects (e.g., parasitic wasps, bees, ladybirds, lacewings and predaceous bugs), birds, fish and mammals [10,11,12]. Flonicamid works by rapidly inhibiting pest feeding behavior shortly after applications, ultimately leading to mortality and providing long-lasting control [10]. Initially classified under mode of action group 9C (chordotonal organs TRPV channel modulators) by the Insecticide Resistance Action Committee (IRAC), flonicamid is now categorized under group 29 (chordotonal organ nicotinamidase inhibitors) [13]. This unique mode of action makes flonicamid an effective alternative for managing resistant *Lygus* populations compared to conventional insecticides (e.g., pyrethroids, organophosphates, carbamates and neonicotinoids) by significantly reducing potential cross-resistance and minimizing non-target effects [10,12]. Flonicamid has been widely adopted for *L. hesperus* control in Arizona since 2005, where the population of natural enemies has been effectively maintained and high crop yields were achieved even with minimal spray of flonicamid [8]. Field trials demonstrated that flonicamid application at rates of 0.036 to 0.088 lb active ingredient per acre (50–100 g ai/ha) exhibited potent efficacy against *Lygus* adults and nymphs for up to 10 days after treatment [14]. Under moderate pest pressure, its performance was comparable to standard neonicotinoid and pyrethroid-neonicotinoid combination treatments [14].

Insect detoxification enzymes, such as cytochrome P450 monooxygenases (P450s), esterases and glutathione-S-transferase (GST), play a crucial role in metabolic detoxification by catalyzing reactions (e.g., oxidation, hydroxylation and deamination reactions), which modify the chemical structure of exogenous substances [15,16]. This process enables insects to withstand insecticide exposure by converting toxic, insoluble compounds into water-soluble excretable forms [17,18,19]. Metabolic resistance, often driven by elevated detoxifying enzyme activity, is the primary mechanism conferring resistance to various chemical classes [15]. For instance, resistance to neonicotinoid insecticides in *L. lineolaris* has been attributed to increased P450 activity and the overexpression of P450 monooxygenase genes [20]. Therefore, understanding how detoxification enzymes interact with flonicamid is essential for developing sustainable pest management strategies.

Flonicamid has been a recommended component of *L. hesperus* management strategies in California since 2005 and continues to be an effective tool for controlling this pest in the southwestern agricultural system [21,22,23]. Similarly, it was used to control *L. lineolaris* populations in Mississippi from 2015 to 2021 [24,25], though it was removed from pest management recommendations in 2022 [26]. In this study, we evaluated the comparative toxicity of flonicamid (Carbine 50WG) to two laboratory-reared populations of *L. lineolaris* and *L. hesperus* and examined potential detoxification mechanisms in both species. Two bioassay methods (spray and dipping assay) on the third-instar nymphs and adults were employed to assess toxicity via different exposure routes. Additionally, synergistic experiments and enzymes activity assays were conducted to examine the role of detoxification enzymes, including P450, esterase and GST. These findings provide valuable insights into the practical application of flonicamid for *Lygus* management in the field.

## 2. Materials and Methods

### 2.1. Insect Populations and Insecticides

Tarnished plant bug (TPB, *Lygus lineolaris*) and western tarnished plant bug (WTPB, *Lygus hesperus*) populations were maintained in the laboratory without exposure to any pesticides since 2014. Both species were reared on fresh broccoli, which was replaced every two weeks, under controlled conditions of 28 ± 1 °C, 70 ± 5% relative humidity and 12:12 h light/dark photoperiod. The insecticide Carbine TM 50 WG (50% flonicamid active ingredient) used in this study was obtained from FMC Corporation Agricultural Products Group (Philadelphia, PA, USA).

### 2.2. Spray and Dipping Bioassay

The spray bioassay followed a previously established protocol for evaluating flonicamid toxicity on the third-instar nymphs and adults of *L. lineolaris* and *L. hesperus* [20,27]. Four to seven concentrations of flonicamid solutions (ranging 10–3000 mg/L) were prepared by dissolving flonicamid in deionized water and serially diluting to achieve the desired concentrations. To ensure acceptable control mortality, fresh green beans (washed in 1% bleach solution) were replaced every two or three days. The third-instar nymphs or adults of *L. lineolaris* and *L. hesperus* were placed into 500 mL round, wide-mouth polypropylene plastic cups (D × H: 9.3 × 10 cm) with fabric mesh covered holes (5.0 cm diameter) on both the lid and bottom. Each cup contained 10 third-instar nymphs or adults, and two 7–8 cm long green beans placed at the bottom. A custom-modified spray tower, made of Plexiglass to fit into a fume hood and equipped with an original spray nozzle of a Potter Spray Tower (Burkard Scientific Ltd., Uxbridge, UK) was used to apply treatment. The spray system delivered consistent air pressure (69 kPa or 10 psi) and spray distance of 22 cm, mimicking the performance of a standard Potter Spray Tower. For each treatment, 0.5 mL of flonicamid solution was sprayed directly into each cup, covering the inner wall, green beans and insects. The same concentration was reapplied when beans were replaced on day 2 and 5. For the dipping assay, each green bean was immersed into varying concentrations of flonicamid for 10 s, air dried for 30 s and then placed in the cups containing bugs. Both assays were conducted with three to four replicates per insect batch; a total of two or three batches were used in the bioassays. A control was included by treating bugs with deionized water. After treatment, all cups were placed in an environmental incubator set at 28 ± 1 °C, 70 ± 5% RH, and a 12:12 (L:D) photoperiod. Dead *Lygus* nymphs or adults were recorded on day 2, 5 and 7 for all experiments. Individuals were considered dead if they were unable to walk or fly when gently touched with brush.

### 2.3. Synergistic Experiments

To assess the involvement of detoxification enzymes in flonicamid metabolism, synergist bioassays were performed using three enzyme inhibitors: triphenyl phosphate (TPP), diethyl maleate (DEM) and piperonyl butoxide (PBO). A 0.5 mL solution of each synergist (0.5% TPP, 0.5% DEM, or 0.25% PBO) was applied to adult *L. lineolaris* and *L. hesperus* by spraying bugs two hours prior to flonicamid exposure [27]. Subsequently, the spray bioassay was conducted as described in Section 2.2. The synergism ratio (SR) was calculated as the ratio of the lethal concentration (LC_50_) value from *Lygus* flonicamid-only treatments to the LC_50_ value from the combined flonicamid and synergist.

### 2.4. Detoxification Enzyme Activity Assays

#### 2.4.1. Chemicals

The following chemicals used in the enzyme activity were purchased from Sigma-Aldrich (St. Louis, MO, USA): protease inhibitor (cocktail tablets), α-naphthyl acetate, α-naphthol, sodium lauryl sulphate, fast blue B salt, 1-chloro-2,4-dinitrobenzene (CDNB), L-glutathione reduced (GSH), umbelliferone (7-hydroxycoumarin), 7-ethoxycoumarin (7-EC), acetonitrile and Trizma base buffer.

#### 2.4.2. Enzyme Preparation

The activities of three major detoxification enzymes (esterase, GST and P450) were measured in *L. lineolaris* and *L. hesperus* adults after 7-day exposure to a flonicamid concentration at approximately the LC_50_ value determined by the bioassays. Six replicates (n = 2/rep) were homogenized (Homogenizer, Thomas Scientific, Swedesboro, NJ, USA) in 500 μL of ice-cold sodium phosphate buffer (0.1 M, pH 7.2) containing 0.1% Triton X-100 and protease inhibitor. Homogenates were centrifuged (10,000× *g*, 15 min, 4 °C) and the resulting supernatant was used for enzyme assays. Undiluted supernatant was used for P450 assays, 4-fold dilutions for protein and GST assays, and 20-fold dilutions for esterase assays using homogenization buffer without Triton X-100. Total protein concentration was quantified using a Bradford protein assay kit with a bovine serum albumin standard [28] (Thermo Scientific, Waltham, MA, USA). All enzyme and protein assays were performed in three replicates using a microplate reader (Agilent BioTek Synergy H1 Multimode Microplate Reader, Winooski, VT, USA).

#### 2.4.3. Esterase Activity Assays

Esterase activity was determined using α-naphthyl acetate as substrates, following modified methods from Dorman et al. and Zhu et al. [29,30]. In each well, 10 μL of enzyme solution (4-fold dilution in 0.1 M sodium phosphate buffer, pH 7.2) was mixed with 135 μL of 0.3 mM α-naphthyl acetate solution. After incubation at 37 °C for 30 min, the reaction was stopped by adding 50 μL of fast blue B salt (3 mg/mL) in 5% sodium lauryl sulphate solution. Absorbance was measured at 600 nm after a 15 min incubation at room temperature. Esterase activity was calculated based on the standard linear relationship established using α-naphthol per minute per milligram of protein.

#### 2.4.4. Glutathione S-Transferase (GST) Activity Assays

GST activity was assessed using CDNB as the substrate, following modified protocols from Zhu et al. [29]. The reaction mixture (120 μL) contained 10 μL of the enzyme solution, 10 μL of 2 mM CDNB, 50 μL of 10 mM GSH, and 50 μL of 0.1 M sodium phosphate buffer (pH 7.5). Absorbance at 340 nm was recorded every 10 s for 10 min. GST activity was calculated based on the extinction coefficient of 5.3 mM^−1^ cm^−1^ for CDNB [31].

#### 2.4.5. Cytochrome P450 Monooxygenase (P450) Assays

P450 activity was measured by the CYP450-mediated O-deethlylation of 7-exthoxycoumarin (7-EC) to 7-hydroxycoumarin reaction, following previously established protocol [20,27]. In each well of a black 96-well flat-bottom microplate, 40 µL of undiluted enzyme extract was mixed with 76 μL of 0.1 M sodium phosphate buffer (pH 7.2) and 4 μL of 8 mM 7-EC in 95% ethanol as a substrate. The plate was incubated at 37 °C at 200 rpm for 4 h in an incubator shaker (Thermo Scientific, Waltham, MA, USA). The reaction was terminated by adding 120 μL of 50% (*v*/*v*) acetonitrile in 50 mM Trizma-base buffer (pH = 10). Fluorescence of 7-hydroxycoumarin was measured at 460 nm while exciting at 360 nm. P450 activity (7-EC-O-deethylation, ECOD) was determined based on the 7-hydroxycoumarin standard curve, and the protein concentration and activity were expressed as pmol/min of 7-hydroxycoumarin formed per mg.

### 2.5. Data Analysis

All experiments were conducted with three replicates, and the results were presented as means ± standard error (SE). LC_50_ values and 95% confidence intervals were determined using Probit analysis with SPSS software (version 19.0, SPSS Inc., Chicago, IL, USA). Significant differences in LC_50_ values among *Lygus* populations were identified based on non-overlapping 95% confidence intervals (CIs). Chi-square testing was used to assess the linearity of dose–mortality response in Pearson Goodness of Fit Test. The 95% CIs were calculated using standard Probit methods Enzymatic activity data were plotted using JMP software (version 17.0), with statistical significance determined using one-way analysis of variance with Tukey’s HSD test and significant values were set at *p* < 0.05.

## 3. Results

### 3.1. Toxicity of Flonicamid to L. lineolaris and L. hesperus

**Figure 1:**
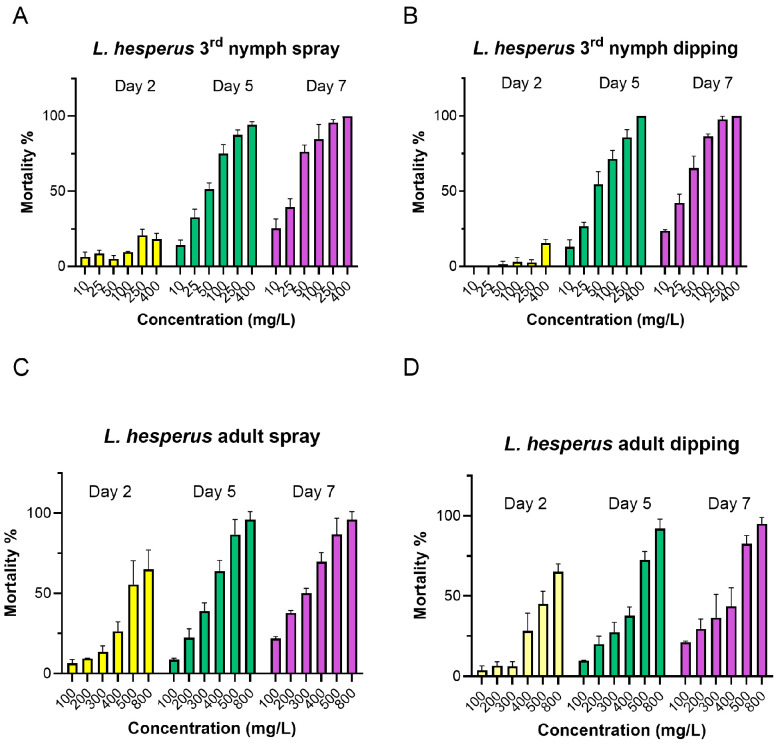
Mortality of the third-instar nymph and adults of *L. hesperus* treated with flonicamid at day 2, 5 and 7 in spray and dipping bioassays. Flonicamid concentrations ranged from 10 to 400 mg/L for third-instar nymphs ((**A**): spray; (**B**): dipping), and 100 to 800 mg/L for adults ((**C**): spray; (**D**): dipping). Mortality data are presented as means ± SE; each treatment was conducted with three to four replicates, with all data obtained from three independent *L. hesperus* colonies.

The toxicity of flonicamid to *L. lineolaris* and *L. hesperus* was evaluated using both spray and dipping bioassays. Third-instar nymphs and adults of *L. hesperus* were exposed to flonicamid at concentrations ranging from 10 to 400 mg/L and 100 to 800 mg/L, respectively (Figure 1). Control mortality remained consistently below 5% across all assays. Mortality was recorded on day 2, 5 and 7, and minimal or no mortality was observed on day 2, but a concentration-dependent increase in mortality occurred on day 5 and 7 (Figure 1). In the spray bioassay, the LC_50_ value on day 5 ranged from 25.89 to 49.59 mg/L for third-instar nymphs and from 274.83 to 370.18 mg/L for adults (Table 1 and Figure 1). In the dipping bioassay, LC_50_ values were slightly higher, ranging from 34.67 to 58.22 mg/L for third-instar nymphs and 334.51 to 441.21 mg/L for adults. By day 7, the LC_50_ value in the spray assay decreased to 20.72 mg/mL for nymphs and 226.76 mg/L for adults, while in the dipping assay, they were 29.59 mg/mL and 302.61 mg/L, respectively. These results indicated an increase in toxicity over time, though there was no significant difference in LC_50_ value between day 5 and 7. Additionally, no significant difference in toxicity was observed between spray and dipping methods; however, third-instar nymphs exhibited significantly higher sensitivity than adults in both bioassays (Table 1 and Figure 1).

**Figure 2:**
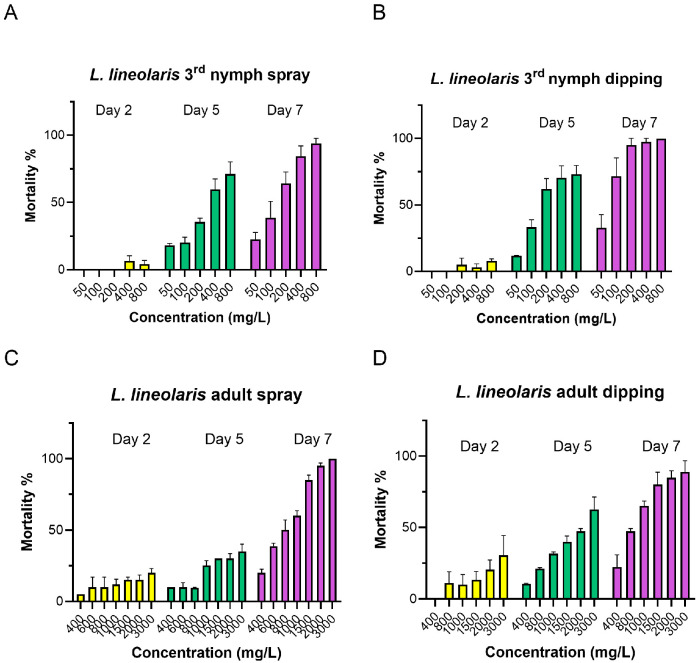
Mortality of the third-instar nymphs and adults of *L. lineolaris* treated with flonicamid at day 2, 5 and 7 in spray and dipping bioassay. Flonicamid concentrations ranged from 50 to 800 mg/L for third-instar nymph ((**A**): spray; (**B**): dipping), and 200 to 3000 mg/L for adults ((**C**): spray; (**D**): dipping). Mortality data are expressed as means ± SE; each treatment was conducted with three to four replicates, with all data obtained from three independent *L. lineolaris* colonies.

Compared to *L. hesperus*, *L. lineolaris* exhibited greater tolerance to flonicamid in both bioassays. Third-instar nymphs were exposed to concentrations ranging 50–800 mg/L, while adults were exposed to 400–3000 mg/L (Table 2 and Figure 2). On day 5, LC_50_ values for third-instar nymphs ranged from 305.02 to 522.74 mg/L in the spray assay and from 239.07 to 467.12 mg/L in the dipping assay. By day 7, the LC_50_ value decreased significantly to 105.0 mg/L in the spray assay and 130.12 mg/L in the dipping assay, showing a 3.79- or 2.68-fold increase in sensitivity, respectively, compared to day 5 (Table 2 and Figure 2). For *L. lineolaris* adults, mortality reached only 60% at the highest tested concentration of 3000 mg/L on day 5 in both bioassays. Therefore, the LC_50_ value for adults could only be calculated for day 7, ranging from 463.26 to 786.85 mg/L in the spray assay and from 547.73 to 949.36 mg/L in the dipping assay (Table 2 and Figure 2). Overall, *L. lineolaris* exhibited significantly higher LC_50_ values than *L. hesperus*, indicating 5.1- to 4.4-fold lower susceptibility in third-instar nymphs and 2.7- to 2.5-fold lower susceptibility in adults for spray and dipping bioassays, respectively, on day 7 (Table 1 and Table 2).

### 3.2. Synergistic Effects of Enzyme Inhibitors on Flonicamid Toxicity

**Figure 3:**
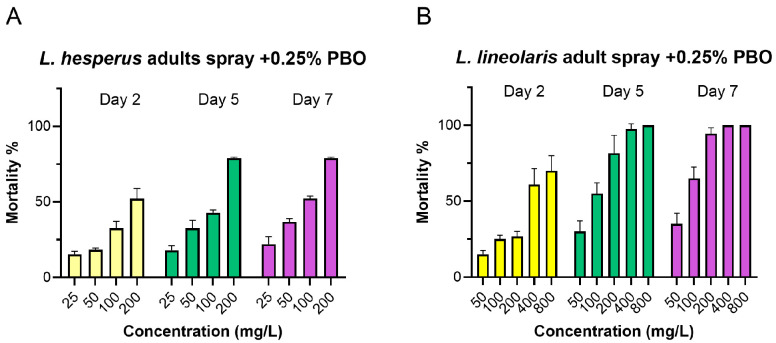
Mortality of adult *L. hesperus* (**A**) and *L. lineolaris* (**B**) at day 2, 5, and 7 after flonicamid treatment at a range of 25–200 mg/L for *L. hesperus* and 50–800 mg/L for *L. lineolaris*. Flonicamid treatment was conducted two hours after prior treatment with 0.25% PBO. Mortality data are presented as means ± SE; each treatment was conducted with four to six replicates, with all data obtained from three independent *Lygus* colonies.

The presence of PBO significantly enhanced the toxicity of flonicamid. In *L. hesperus* adults, the effective concentration range decreased to 25–200 mg/L with PBO, compared to 100–800 mg/L without it. In *L. lineolaris,* the range decreased to 50–800 mg/L with PBO, compared to 400–3000 mg/L initially (Figure 1 and Figure 3). PBO (0.25%) exhibited a significant synergistic effect with synergistic ratios (SR) of 3.26 and 2.77 for *L. lineolaris* at day 5 and 7, respectively, and 5.98 and 7.58 for *L. hesperus* at day 5 and 7 (Table 3). In contrast, DEM showed no significant synergistic effect, with SR values of 0.99 for *L. lineolaris* and 1.10 for *L. hesperus* adults on day 5. Similarly, TPP did not exhibit significant synergism, with SR values of 0.95 for *L. hesperus* and 1.18 for *L. lineolaris* at day 7 (Table 3 and Figure 3).

### 3.3. Detoxification Enzyme Activity Following Exposure to Sublethal Dose of Flonicamid

**Figure 4:**
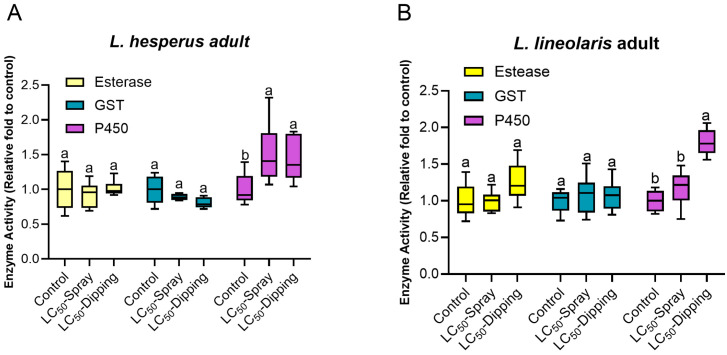
Enzyme activities of esterase, GST and P450 in adult *L. lineolaris* (**A**) and *L. hesperus* (**B**) following exposure to a sublethal dose of flonicamid (400 mg/L for *L. hesperus*, 800 mg/L for *L. lineolaris*) via spray and dipping bioassays. Enzyme activities are expressed as the relative ratio to the ddH_2_O control. Box plots represent the 10th to 90th percentiles, with median indicated. Within each enzyme group, bars labeled with different lowercase letters are significantly different (*p* < 0.05, one-way analysis of variance with Tukey’s HSD test).

Detoxification enzyme activities were evaluated at day 7 post-exposure to LC_50_ value of flonicamid. In *L. hesperus* adults (400 mg/L), P450 activity (df = 2, 14, F = 4.75, *p* = 0.03) significantly increased by 1.57-fold in the spray bioassay and 1.43-fold in the dipping bioassay compared to the control. Similarly, in *L. lineolaris* adults (800 mg/L), P450 activity (df = 2, 14, F = 9.06, *p* = 0.003) significantly increased 1.70-fold in the dipping bioassay, though the increase in spray assay (1.18-fold) was not significant. No significant changes were observed in esterase (*L. hesperus*: df = 2, 14, F = 0.32, *p* = 0.73; *L. lineolaris*: df = 2, 11, F = 2.64, *p* = 0.11) or GST(*L. hesperus*: df = 2, 14, F = 3.09, *p* = 0.08; *L. lineolaris*: df = 2, 14, F = 0.21, *p* = 0.81) activity in either species following exposure to a sublethal dose of flonicamid compared to control (ddH_2_O) in both spray and dipping bioassays (Figure 4). 

## 4. Discussion

In this study, flonicamid exhibited significantly higher toxicity to third-instar nymphs and adults of *L. hesperus* compared to *L. lineolaris* in both spray and dipping bioassays. This differential susceptibility may help explain why flonicamid remains recommended for *Lygus* control in California [21,22,23], whereas it has been removed from pest management recommendations in Mississippi since 2022 [24,25,26,32,33,34,35,36]. Additionally, our findings showed that the third-instar nymphs of both species were more sensitive to flonicamid than adults, consistent with field results, where flonicamid has been particularly effective against nymph populations [14,37]. Unlike conventional insecticides, flonicamid acts by disrupting insect feeding behavior and leads to delayed mortality. This slow-acting mode of action explains the extended bioassay durations; reliable toxicity estimates were achieved through 5- and 7-day assessment. While developmental delays were not reported in the current study, flonicamid can delay growth effects in whiteflies [38]. Flonicamid toxicity was assessed using two bioassay methods—spraying and dipping—representing combined contact and oral exposure versus oral exposure alone, respectively. No significant differences were observed between the two methods, suggesting that flonicamid primarily acts through ingestion rather than contact.

The toxicity of flonicamid varies widely among different pest species, including aphids, whiteflies and planthoppers. For cotton aphids (*Aphis gossypii*), flonicamid exhibited high potency, with LC_50_ values ranging from 1.43 to 60 mg/L, comparable to other commonly used insecticides [39]. Similarly, for the soybean aphid (*Aphis glycines*), flonicamid toxicity ranked lower than dimethoate and λ-cyhalothrin, but more effective than mineral oil, *Beauveria bassiana*, and spirotetramat [40]. When combined with an entomopathogenic fungi (*Metarhizium anisopliae* (Metschin)), flonicamid exhibited significantly higher toxicity (91.7% mortality within 72 h) to cotton aphids than imidacloprid, nitenpyram, dinotefuran, pyriproxyfen, spirotetramat or flonicamid alone [41]. Flonicamid also demonstrated high toxicity to the adult greenbug (*Schizaphis graminum*) (Day 2, LC_50_ = 5.1 mg/L) and third-instar brown planthoppers (*Nilaparvata lugens*), with LC_50_ values of 94.5, 24.7 and 18.9 mg/L on day 3, 5 and 7, respectively [42]. In contrast, flonicamid showed relatively low toxicity to whiteflies (*Bemisia tabaci* Mediterranean) after 10-day exposures, with LC_50_ values ranging from 95.7 to 1001 mg/L [38]. Compared to these species, the LC_50_ values obtained in this study for *L. hesperus* (156.34–297.78) and *L. lineolaris* (463.46–786.85) (Table 1 and Table 2) on day 7 in spray bioassay were higher than those reported for aphids and planthopper, but comparable to values observed for whiteflies [38]. Interestingly, while flonicamid demonstrated strong activity against third-instar *Lygus* nymphs in our study, its toxicity to *B. tabaci* nymphs was considerably lower (LC_50_: 760 mg/L) [38].

Detoxification enzymes, particularly P450-dependent monooxygenases, are known to play a crucial role in helping insects counteract against exogenous chemicals [15,16]. The synergist PBO, a known P450 inhibitor, enhances the efficacy of various insecticides (e.g., pyrethroids and neonicotinoids). In our study, synergism experiments revealed that flonicamid toxicity significantly increased in the presence of PBO. Moreover, sublethal exposure to flonicamid induced increased P450 enzyme activity in both *Lygus* species, indicating that P450s are involved in flonicamid detoxification. In contrast, no significant synergistic effects were observed with DEM or TPP, which inhibit GST and esterases, respectively. These findings are consistent with results in the red imported fire ant (*Solenopsis invicta*), where P450 enzymes were also implicated in flonicamid detoxification [43]. Overall, our results suggest that cytochrome P450 monooxygenases are primary enzymes responsible for the metabolic detoxification of flonicamid, thereby reducing its toxicity over time.

The present study revealed that *L. hesperus* was significantly more susceptible to flonicamid than *L. lineolaris*. Both *Lygus* populations used in this study had been maintained under laboratory conditions for 7–8 years without any exposure to insecticides. However, the *L. lineolaris* colony was originally collected from the Mississippi Delta, where field populations have historically experienced high insecticide selection pressure, with an average of 4.75 and up to 12 applications per year [27]. Such intensive insecticide use may have accelerated resistance development in this population [27]. Previous studies have shown that field-collected *L. lineolaris* populations resistant to pyrethroids and neonicotinoids often exhibited elevated P450 activities, which likely contribute to reduced susceptibility to flonicamid [20,27,44].

Flonicamid is a novel systemic insecticide with a unique mode of action that distinguishes it from conventional insecticides and reduces the potential for cross-resistance. Its primary effect is to disrupt feeding behavior by targeting physiological pathways essential for insect feeding and survival. Specifically, flonicamid selectively targets insect chordotonal organs, crucial sensory structures involved in detecting mechanical vibrations and proprioception. Flonicamid functions as a pro-insecticide, with its bioactive metabolite 4-trifluoromethlynicotinamide (TFNA-AM) disrupting chordotonal organ function in a manner similar to group 9 insecticides [9,11]. However, unlike afidopyropen, which modulates the transient receptor potential vanilloid (TRPV) channel, flonicamid does not interact with the TRPV channel in heterologous systems or with [^3^H]-afidopyropen binding sites. Recent findings suggest that flonicamid targets nicotinamidase (Naam), a key enzyme involved in nicotinamide metabolism [45]. Thus, while afidopyropen acts as TRPV channel modulator, flonicamid operates as a nicotinamide inhibitor, representing a novel mechanism of action effective against piercing–sucking pests, such as peach aphids *M. persicae* [11]. Flonicamid is classified as an anti-feeding insecticide; electrical penetration graph (EPG) experiments have demonstrated that flonicamid significantly reduced phloem feeding activity, honeydew excretion, and fecundity in pests like the brown planthopper *Nilaparvata lugens* [42]. The EPG system incorporates the insect and its feeding substrate into an electrical circuit, generating waveform signals that correspond to distinct penetration and feeding behaviors [46,47]. Moreover, inward rectifier potassium (Kir) channels may also be targets of flonicamid, contributing to its anti-feeding activity [48].

## 5. Conclusions

This study demonstrated that flonicamid exhibited greater toxicity to *L. hesperus* than to *L. lineolaris* in both spray and dipping bioassays. No significant differences were observed between the two bioassay methods, suggesting that flonicamid acts primarily as a feeding blocker rather than contact toxicity. Additionally, nymphs of both *Lygus* species were significantly more susceptible than adults, consistent with field observations that flonicamid is particularly effective against immature stages. These findings indicate that flonicamid may still be a viable option for early-season control of *L. lineolaris* nymphs, especially when generations are not overlapping. Synergism assays with PBO and elevated P450 activity following sublethal exposure suggest that cytochrome P450 enzymes are involved in the detoxification of flonicamid. Future studies using EPG and electrophysiological methods will be valuable to further elucidate the anti-feeding effects of flonicamid in *Lygus* species and optimize its use in pest management programs.

## Figures and Tables

**Table 1:** Toxicity of flonicamid against third-instar nymphs and adults of *L. hesperus* at day 5 and 7 in spray and dipping bioassays.

Species (*L. hesperus*)	Days	n	Slope	LC_50_ (μg/mL) ^a^	95% Confidence Limits (μg/mL) ^a^	χ^2 b^	df ^c^	*p*
3rd nymph	Day 5	279	1.21 ± 0.14	36.25	25.89–49.59 cd	5.63	5	0.35
(Spray)	Day 7	279	1.34 ± 0.16	20.72	14.53–27.96 d	7.88	5	0.16
3rd nymph	Day 5	276	1.58 ± 0.19	45.74	34.67–58.22 c	5.98	4	0.20
(Dipping)	Day 7	276	1.92 ± 0.23	29.59	22.48–36.98 cd	3.89	4	0.42
Adult (Spray)	Day 5	274	3.37 ± 0.59	316.35	274.83–370.18 ab	3.54	4	0.32
	Day 7	274	2.18 ± 0.53	226.76	156.34–297.78 b	1.70	4	0.64
Adult (Dipping)	Day 5	272	3.37 ± 0.59	376.91	334.51–441.21 a	2.58	4	0.28
	Day 7	272	2.51 ± 0.62	302.61	224.83–414.09 ab	2.75	4	0.25

^a^ LC_50_ values and 95% confidence intervals were determined through Probit analyses using SPSS software. Different letters indicate significant difference from each other through nonoverlap of 95% CIs. ^b^ Chi-square testing was used to assess the linearity of dose–mortality response in Pearson Goodness of Fit Test. ^c^ Degrees of freedom.

**Table 2:** Toxicity of flonicamid against third-instar nymphs and adults of *L. lineolaris* at day 5 and 7 in spray and dipping bioassay.

Species (*L. lineolaris*)	Days	n	Slope	LC_50_ (μg/mL) ^a^	95% Confidence Limits (μg/mL) ^a^	χ^2 b^	df ^c^	*p*
3rd nymph	Day 5	250	1.51 ± 0.19	398.25	305.02–522.74 b	1.53	5	0.91
(Spray)	Day 7	250	2.18 ± 0.53	105.00	70.43–140.44 c	3.98	5	0.55
3rd nymph	Day 5	217	1.39 ± 0.23	348.11	239.07–467.12 ab	2.06	5	0.84
(Dipping)	Day 7	217	2.44 ± 0.38	130.12	85.98–169.98 c	5.40	5	0.25
Adult (Spray)	Day 7	302	2.15 ± 0.37	620.90	463.26–786.85 ab	0.93	6	0.98
Adult (Dipping)	Day 7	298	2.11 ± 0.35	742.13	547.73–949.36 a	1.19	5	0.95

^a^ LC_50_ values and 95% confidence intervals were determined through Probit analyses using SPSS software. Different letters indicate significant difference from each other through nonoverlap of 95% CIs. ^b^ Chi-square testing was used to assess the linearity of dose–mortality response in Pearson Goodness of Fit Test. ^c^ Degrees of freedom.

**Table 3:** Toxicity of flonicamid against adult *L. lineolaris* and *L. hesperus* with synergists triphenyl phosphate (TPP), diethyl maleate (DEM) and piperonyl butoxide (PBO) at day 5 and 7 post-treatment.

Species	Days	n	Slope	LC_50_ (μg/mL) ^a^	95% Confidence Limits (μg/mL) ^a^	χ^2 b^	df ^c^	*p*	SR ^d^
*L. hesperus*	Day 5	274	3.37 ± 0.59	316.35	274.83–370.18	3.54	4	0.32	--
	Day 7	274	2.18 ± 0.53	226.76	156.34–297.78	1.70	4	0.64	--
	+PBO (Day 5)	186	1.95 ± 0.45	97.18	60.09–138.82 *	1.02	3	0.62	3.26
	+PBO (Day 7)	186	1.76 ± 0.44	81.94	43.12–120.83 *	0.28	3	0.87	2.77
	+DEM (Day 5)	246	6.99 ± 1.31	320.12	281.96–356.57	0.85	4	0.65	0.99
	+DEM (Day 7)	246	5.83 ± 1.16	295.14	251.83–333.98	0.15	4	0.93	0.77
	+TPP (Day 5)	232	2.89 ± 0.36	288.56	248.14–331.82	0.74	4	0.58	1.10
	+TPP (Day 7)	232	2.59 ± 0.49	238.13	196.14–342.28	0.64	4	0.76	0.95
*L. lineolaris*	Day 7	302	2.15 ± 0.37	620.90	463.26–786.85	0.93	6	0.98	--
	+PBO (Day 5)	200	2.59 ± 0.46	103.78	73.46–137.16 *	4.20	3	0.24	5.98
	+PBO (Day 7)	200	3.75 ± 0.78	82.02	62.33–103.23 *	0.91	3	0.64	7.58
	+DEM (Day 7)	192	2.53 ± 0.48	800.36	690.49–1263.61	2.81	4	0.59	0.78
	+TPP (Day 7)	202	2.36 ± 0.43	523.93	376.61–684.75	3.09	4	0.38	1.18

^a^ LC_50_ values and 95% confidence intervals were determined through Probit analyses using SPSS software. * Indicates significant difference in the responses of flonicamid + synergist compared to flonicamid only. ^b^ Chi-square testing was used to assess the linearity of dose–mortality response in Pearson Goodness of Fit Test. ^c^ Degrees of freedom. ^d^ Synergist ratio calculated by dividing LC_50_ of the flonicamid by LC_50_ value of flonicamid + synergist.

## Data Availability

The data presented in this study are available on request from the corresponding author due to restriction related to privacy.
